# NMDA Receptors in GABAergic Synapses during Postnatal Development

**DOI:** 10.1371/journal.pone.0037753

**Published:** 2012-05-25

**Authors:** Csaba Cserép, Eszter Szabadits, András Szőnyi, Masahiko Watanabe, Tamás F. Freund, Gábor Nyiri

**Affiliations:** 1 Laboratory of Cerebral Cortex Research, Department of Cellular and Network Neurobiology, Institute of Experimental Medicine, Hungarian Academy of Sciences, Budapest, Hungary; 2 Department of Anatomy, Hokkaido University School of Medicine, Sapporo, Japan; The Research Center of Neurobiology-Neurophysiology of Marseille, France

## Abstract

GABA (gamma-aminobutyric-acid), the main inhibitory neurotransmitter in the adult brain, exerts depolarizing (excitatory) actions during development and this GABAergic depolarization cooperates with NMDARs (N-methyl-D-aspartate receptors) to drive spontaneous synchronous activity (SSA) that is fundamentally important for developing neuronal networks. Although GABAergic depolarization is known to assist in the activation of NMDARs during development, the subcellular localization of NMDARs relative to GABAergic synapses is still unknown. Here, we investigated the subcellular distribution of NMDARs in association with GABAergic synapses at the developmental stage when SSA is most prominent in mice. Using multiple immunofluorescent labeling and confocal laser-scanning microscopy in the developing mouse hippocampus, we found that NMDARs were associated with both glutamatergic and GABAergic synapses at postnatal day 6–7 and we observed a direct colocalization of GABA_A_- and NMDA-receptor labeling in GABAergic synapses. Electron microscopy of pre-embedding immunogold-immunoperoxidase reactions confirmed that GluN1, GluN2A and GluN2B NMDAR subunits were all expressed in glutamatergic and GABAergic synapses postsynaptically. Finally, quantitative post-embedding immunogold labeling revealed that the density of NMDARs was 3 times higher in glutamatergic than in GABAergic synapses. Since GABAergic synapses were larger, there was little difference in the total number of NMDA receptors in the two types of synapses. In addition, receptor density in synapses was substantially higher than extrasynaptically. These data can provide the neuroanatomical basis of a new interpretation of previous physiological data regarding the GABA_A_R-NMDAR cooperation during early development. We suggest that during SSA, synaptic GABA_A_R-mediated depolarization assists NMDAR activation right inside GABAergic synapses and this effective spatial cooperation of receptors and local change of membrane potential will reach developing glutamatergic synapses with a higher probability and efficiency even further away on the dendrites. This additional level of cooperation that operates within the depolarizing GABAergic synapse, may also allow its own modification triggered by Ca^2+^-influx through the NMDA receptors.

## Introduction

Spontaneous synchronous activity (SSA) – also known as giant depolarizing potential/GDP *in vitro*, or developmental sharp wave *in vivo* [1] – is a fundamental feature of developing networks, it is conserved through evolution, and can be observed throughout the entire central nervous system [2]. Synaptic transmission and this correlated synchronous activity of neuronal ensembles are fundamental for proper circuit formation in the developing brain [2], [3]. This is achieved by the depolarizing actions of GABA_A_-receptors and activation of N-methyl-D-aspartate ionotropic glutamate receptors (NMDARs) during early postnatal days [4], [5].

NMDARs are essential contributors to fast glutamatergic excitatory synaptic transmission, as well as to several forms of synaptic plasticity in the adult brain; however, they also play critical roles during the development of neuronal networks. Blockade of NMDARs at this age greatly reduces synchronous network activity both in neocortex and in hippocampus [6]–[8], and leads to a series of severe neuromorphological and behavioral deficits [9]–[12]. Furthermore, NMDAR activation is also essential for the recruitment of AMPA (2-amino-3-(5-methyl-3-oxo-1,2- oxazol-4-yl)propionic acid) receptors into silent synapses and for other types of Ca^2+^-dependent synaptic plasticity during development [13]–[16].

The contribution of GABA_A_Rs and NMDARs to the first synapse-driven synchronous activities is essential [4], [5]. Previous findings also showed that after the recruitment of AMPA receptors to glutamatergic synapses, AMPA receptors may also contribute to the SSA, but their blockade has little influence on it [4], [6], [17]. In the first postnatal week, GABA exerts a complex depolarizing (excitatory/shunting inhibitory) action [18], and this GABAergic depolarization is sufficient – even in the absence of functional AMPA receptors - to remove the Mg^2+^-block from the NMDARs, thus leading to a postsynaptic calcium influx that is required for the developmentally relevant synchronous network activity, and the recruitment of AMPA receptors into silent synapses. Previously, NMDARs responsible for the GABA_A_R-NMDAR cooperation were thought to be present exclusively in glutamatergic synapses [19]. However, several studies concluded that the emergence of GABAergic synapses precedes that of glutamatergic ones [20]–[22], therefore it is not clear whether NMDARs at those glutamatergic synapses could be the only NMDARs that are responsible for the massive GABA_A_R-NMDAR cooperation during early SSAs. Previous physiological studies did not investigate whether these NMDAR currents originated from glutamatergic synapses or from other NMDARs closer to GABAergic synapses. This question is even more prominent in the light of our previous work, in which we proved the presence of NMDARs in GABAergic synapses of the adult brain [23].

NMDAR expression has been extensively studied in the adult brain, and there is substantial knowledge concerning their presence during development. The low-resolution distribution of different NMDAR subunit mRNAs and proteins has been described [24]–[26], and ultrastructural studies proved the presence of NMDAR subunits both synaptically and extrasynaptically [19], [27]. However, in spite of their obvious importance, the precise subcellular distribution of NMDAR subunits during postnatal development is unknown. In this study, we examined the exact membrane distribution of NMDARs at an age, when the first synapse-driven synchronous network activity is maximal (at postnatal day 6–7, P6-7) [6], [7] and GABAergic synapses are still depolarizing. Using confocal laser-scanning microscopy, we showed that NMDARs were associated with glutamatergic and GABAergic synapses and we could observe direct colocalization of GABA_A_ and NMDA receptors in GABAergic synapses. Our electron-microscopic results confirmed that GluN1, GluN2A and GluN2B subunits (formerly known as NR1, NR2A and NR2B subunits, respectively) were all expressed postsynaptically in both glutamatergic and GABAergic synapses at P6-7. Quantitative analysis of high-resolution post-embedding immunogold reactions revealed that the density of NMDARs in glutamatergic synapses was 3 times higher than in GABAergic synapses, and that extrasynaptic membranes expressed these receptors with a density almost two orders of magnitude lower than synapses do. Since GABAergic synapses were about two times larger than glutamatergic ones, there is little difference in the total number of NMDARs in the two types of synapses at this age. Our findings could provide the neuroanatomical basis of a new interpretation of previous physiological data. Here we hypothesize that, during early development, NMDA receptors are essential in developing GABAergic synapses for Ca^2+^-dependent synaptic plasticity, for a sufficiently strong local depolarization to assist the maturation of distant glutamatergic synapses, as well as for driving SSAs.

## Results

### Confocal Microscopy Reveals an Association of NMDARs with Both Glutamatergic and GABAergic Synapses during Development

**Figure 1:**
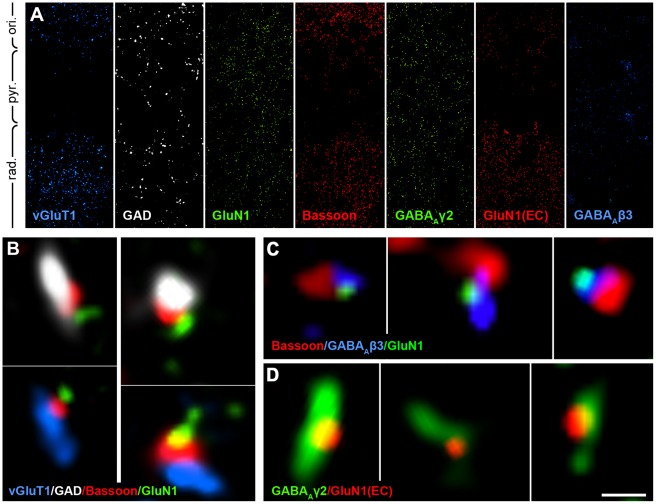
NMDARs are associated with GABAergic and glutamatergic synapses during postnatal development. A , Maximal intensity projection images of three image planes from deconvolved stacks show the general labeling patterns for each staining throughout the strata oriens (ori.), pyramidale (pyr.) and radiatum (rad.) in the CA1 area of the hippocampus. **B–D**, Deconvolved confocal laser-scanning images of quadruple (B), triple (C) and double (D) fluorescent labeling reactions from stratum radiatum of the hippocampal CA1 region. B, In quadruple labelings, vGluT1 (blue) labels glutamatergic axon terminals, GAD65/67 (white) labels GABAergic axon terminals, Bassoon (red) labels presynaptic active zones of both types of synapses. GluN1 (green) is an obligatory NMDAR subunit and it is associated with both types of synapses at P6-7. GABAergic and glutamatergic terminals were sampled from the same image stacks. C, In the triple reaction, Bassoon (red) labels presynaptic active zones, while the labeling of GABA_A_R beta3 subunit (blue) and the obligatory NMDAR subunit GluN1 (green) is overlapping. D, In the double reaction, GABA_A_R gamma2 subunit labeling (green) and the reaction against the extracellular loop of the GluN1 subunit (red) is overlapping as well. The images shown on B, C and D are collected from the stratum radiatum of CA1 area, from single focal planes and the resolution of the imaging was 50 nm50 nm×150 nm/x-y-z voxel size. Scale bar is 50 µm for A, 500 nm for B and 350 nm for C and D.

NMDARs have heterotetrameric structure with two GluN1 subunits and usually with two GluN2 subunits [28]. Since the GluN1 subunit is obligatory for functional NMDARs [29], to localize these receptors, we performed immunofluorescent labeling experiments with antibodies against this subunit. Synapses were labeled with an antibody against Bassoon, a presynaptic active zone protein. Glutamatergic and GABAergic terminals were labeled with anti-type 1 vesicular glutamate transporter (vGluT1) and an anti-glutamate decarboxylase 65/67 (GAD65/67) antibody, respectively. The overall staining pattern in different hippocampal layers of the CA1 area can be seen in Fig. 1A. During development, numerous glutamatergic and GABAergic synapses are present in stratum radiatum of the hippocampal CA1 region, therefore we investigated this area. vGluT1 and GAD65/67 staining appeared as a non-overlapping punctate labeling pattern, showing the distribution of glutamatergic and GABAergic synapses, respectively. In the quadruple-labeling experiments, we found that Bassoon-positive patches were associated to either vGluT1 or GAD65/67 positive terminals. In several cases, GluN1 subunit labeling was found to be associated to the Bassoon-labeled synapses of both vGluT1-positive (glutamatergic) and GAD65/67-positive (GABAergic) terminals at P6-7 (Fig. 1B; n = 4 animals).

Furthermore, we used confocal laser-scanning microscopy to show direct colocalization of GABA_A_- and NMDA-receptors within the same synapses. First, we used a triple labeling protocol for Bassoon, GluN1, and GABA_A_R beta3 subunit (Fig. 1C, n = 2 animals), and in some cases we observed that labeling for GluN1 and GABA_A_R beta3 subunits overlapped, and were juxtaposed to Bassoon-positive patches. Second, we used double-labeling against the extracellular loop of the GluN1 subunit, combined with GABA_A_R gamma2 subunit (Fig. 1D, n = 2 animals) and we also found a frequent association of the GluN1 subunit with GABA_A_R gamma2 labeling. Although the specificity of this method is high, due to its moderate sensitivity, quantification was not performed. Our results obtained using these fluorescent staining experiments suggested that GluN1 labeling associated with GABA_A_R-positive puncta, which showed the synaptic co-expression of NMDARs and GABA_A_Rs during postnatal development. Although these data suggested a close association of NMDARs to both types of synapses, these receptors could still be either pre- or postsynaptic, as well as peri- or intrasynaptic. Therefore, to obtain data that are more precise, we performed electron microscopic experiments as well.

### Preembedding Immunogold Electron Microscopy Confirms the Presence of Three Types of NMDAR Subunits Postsynaptically in Glutamatergic and GABAergic Synapses during Development

**Figure 2:**
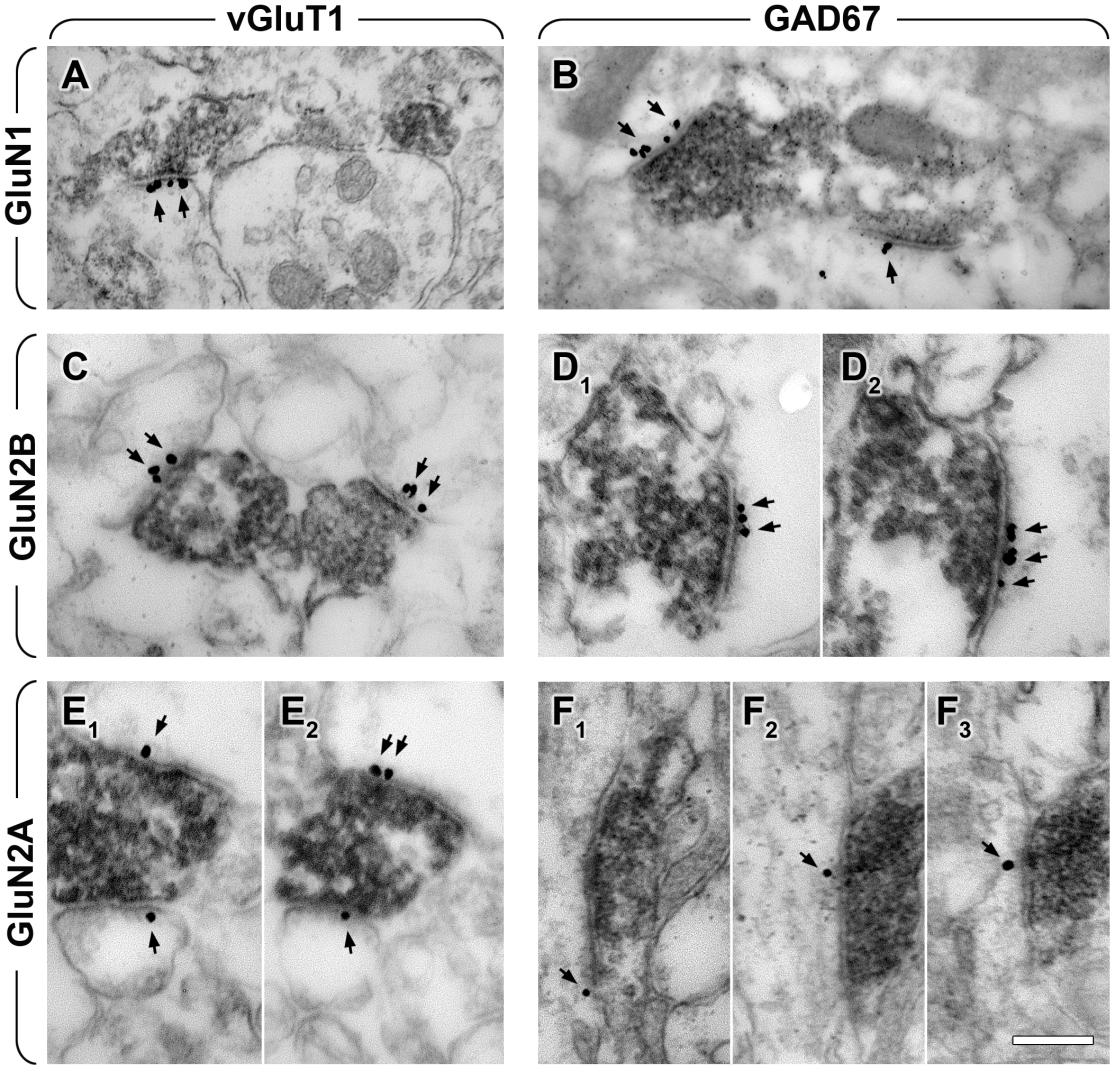
NMDAR subunits are expressed postsynaptically in both GABAergic and glutamatergic synapses at postnatal day 6–7. Electron micrographs show combined immunogold-immunoperoxidase reactions from stratum radiatum of the hippocampal CA1 region. Synapses of vGluT1 positive presynaptic terminals (marked by dark reaction product in A, C, E1 and E2) contain postsynaptic GluN1 (black particles in A, arrows), GluN2B (black particles in C, arrows), and GluN2A subunits (black particles in E1 and E2, arrows). Synapses of GAD67-positive presynaptic terminals (marked by dark reaction product in B, D1, D2, F1, F2 and F3) contain postsynaptic GluN1 (black particles in B, arrows), GluN2B (black particles in D1 and D2, arrows), and GluN2A subunits (black particles on F1, F2 and F3, arrows). Serial images show the same synapse in D1 and D2; E1 and E2; F1, F2 and F3. Scale bar is 300 nm for all images.

**Figure 3:**
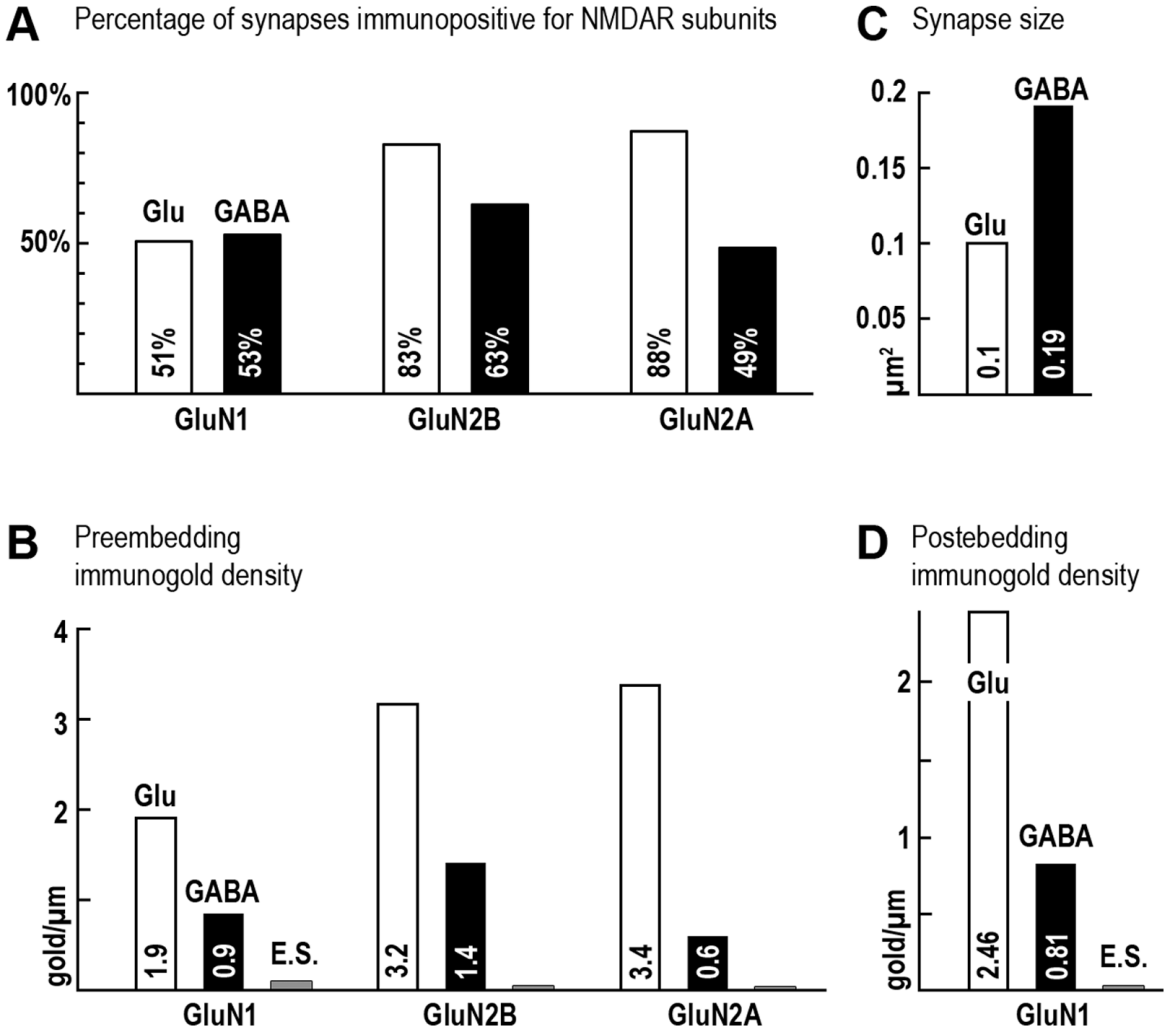
Analysis of the expression of NMDAR subunits in GABAergic and glutamatergic synapses at postnatal day 6–7. A , Percentage of synapses positive for different NMDAR subunits out of all identified glutamatergic (white columns) and GABAergic synapses (black columns). **B**, Linear density of labeling (gold particle/µm) for different NMDAR subunits in glutamatergic synapses (white columns), GABAergic synapses (black columns) and extrasynaptic membranes (grey columns), measured on 100 nm thick electron microscopic sections. The extrasynaptic density of labeling was 0.09, 0.03 and 0.02 immunogold particles/µm for GluN1, GluN2B and GluN2A subunits, respectively. **C**, Size (µm^2^) of glutamatergic (white column) and GABAergic (black column) synapses. Data shown in A, B and C were measured from preembedding experiments. **D**, Density of labeling (gold particle/µm) for GluN1 subunits in glutamatergic synapses (white column), GABAergic synapses (black column) and extrasynaptic (E.S.) membranes (grey column, 0.03 gold particles/µm), measured from quantitative post-embedding experiments.

Since the GABA_A_R and NMDAR dependent synchronous network activity is most prominent around P6-7 in mice [6], [7], we focused our experiments on this developmental stage. We performed immunogold-immunoperoxidase reactions in several combinations. GluN1, GluN2B or GluN2A subunits were labeled with immunogold particles, while glutamatergic and GABAergic terminals were labeled for vGluT1 or GAD67 with the peroxidase method, respectively. Randomly sampled synapses from the stratum radiatum of the CA1 area were examined in each reaction. We found that all three tested subunits of NMDARs were present postsynaptically in both glutamatergic and GABAergic synapses at P6-7 (Fig. 2). Synapses were fully reconstructed from serial electron-microscopic sections from two mice, and at least 51±16% of all glutamatergic (n = 41) and 53±13% of all GABAergic synapses (n = 40) were found to be positive for the GluN1 subunit (Fig. 2A and 2B, Fig. 3A). The linear density of immunolabeling was 1.93±0.03 gold particles/µm in glutamatergic and 0.87±0.29 gold particles/µm in GABAergic synapses, and only 0.09±0.01 gold particles/µm extrasynaptically, on 100 nm thick sections (Fig. 3B, for details on measuring density of immunogold particles, see Materials and Methods). Our data showed that at least 83±3% of all glutamatergic (n = 40) and 63±13% of all GABAergic synapses (n = 40) were positive for the GluN2B subunit (Fig. 2C and 2D_1_–D_2_, Fig. 3A). The linear density of labeling was 3.16±1.01 gold particles/µm in glutamatergic and 1.38±0.34 gold particles/µm in GABAergic synapses, and only 0.03±0.01 gold particles/µm were found extrasynaptically (Fig. 3B). At least 88±8% of all glutamatergic (n = 40) and 49±1% of all GABAergic synapses (n = 43) contained GluN2A subunits (Fig. 2E_1_–E_2_, 2F_1_–F_3_, Fig. 3A). The linear density of the labeling was 3.40±0.81 gold particles/µm in glutamatergic and 0.59±0.28 gold particles/µm in GABAergic synapses, and only 0.02±0.003 gold particles/µm were extrasynaptic (Fig. 3B).

### Quantitative Postembedding Immunogold Method Reveals Only Small Differences in the Number of NMDARs in Glutamatergic and GABAergic Synapses during Development

**Figure 4:**
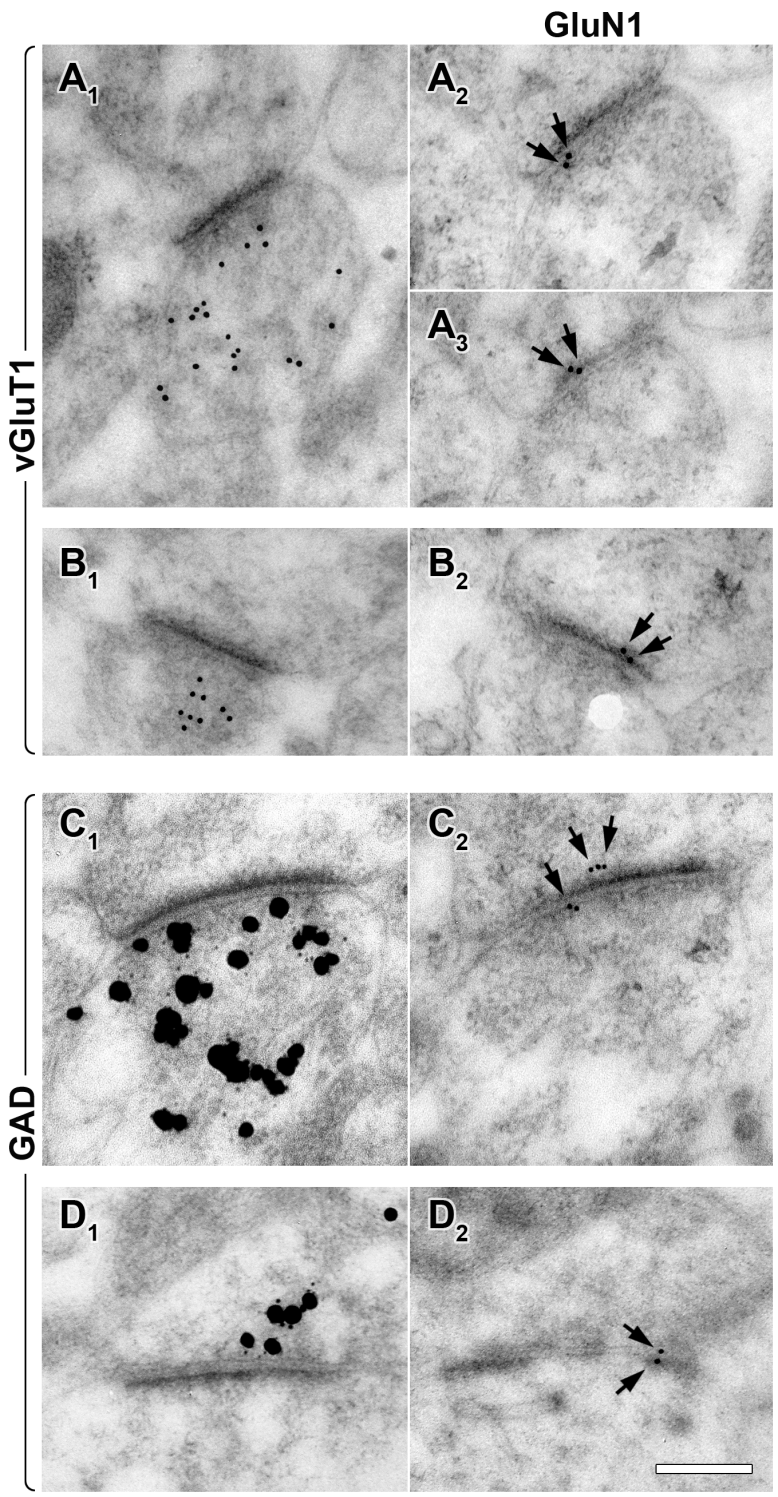
Quantitative post-embedding immunogold labeling reveals NMDAR expression levels in GABAergic and glutamatergic synapses at postnatal day 6–7. The same synapses were reacted with antibodies against different epitopes on adjacent ultrathin sections using the mirror technique. 10 nm immunogold particles label presynaptic glutamatergic (vGluT1 positive) terminals in A1 and B1. Intensified immunogold particles label presynaptic GABAergic (GAD65/67 positive) terminals in C1 and D1. 10 nm immunogold particles (arrows) label GluN1 subunits in A2-3, B2, C2, D2. Note that the postembedding immunoreaction is on the surface of the sections, therefore, the position of gold particles can be on either side of the postsynaptic membrane, even if the labeled epitope is purely postsynaptic. Images from adjacent sections of the same synapses are displayed in A1-3, in B1-2, in C1-2 and in D1-2. Scale bar is 200 nm for all images.

Because of technical limitations, the results of pre-embedding experiments are only semi-quantitative; therefore, we performed post-embedding immunogold labeling to obtain more precise quantitative data on the subcellular distribution of NMDARs. GluN1 staining represents well the distribution of NMDARs, because GluN2 subunits are not transported to the cell-membrane without GluN1 subunits [29], therefore GluN1 is present in all NMDARs. We employed the so-called mirror technique to measure the density of immunogold labeling for the GluN1 subunit. This method enabled us to count these immunogold particles over synapses on the very same sections from two mice and to identify GABAergic and glutamatergic synapses on the mirror sections (for a detailed description, see Materials and Methods). Using this method we could quantitatively compare receptor expression of synaptic and extrasynaptic areas in the P6-7 mice. We randomly collected identified glutamatergic and GABAergic synapses from the stratum radiatum of the CA1 area. NMDAR labeling was detected in both glutamatergic (Fig. 4A–B) and GABAergic synapses (Fig. 4C–D). Our measurements showed that the labeling density was 2.46±0.71 gold particles/µm in glutamatergic (n = 32) and 0.81±0.16 gold particles/µm in GABAergic synapses (n = 28), and only 0.03±0.01 gold particles/µm extrasynaptically (along 103 µm extrasynaptic membrane segment, Fig. 3D). In order to establish the ratio of the absolute number of NMDARs in glutamatergic versus GABAergic synapses, we measured the synaptic areas and multiplied them by the labeling densities measured above in the two types of synapses. For the reliable estimation of synaptic areas, we performed single preembedding immunoperoxidase reactions (on strongly fixed tissue) with antisera against either vGluT1 or GAD67 in two animals, and samples were embedded in Durcupan resin. All synapses were sampled from stratum radiatum. The glutamatergic synaptic area (n = 29) was found to be 0.097±0.013 µm^2^, and GABAergic synapses (n = 32) were 0.186±0.026 µm^2^ (Fig. 3C), i.e. the latter being 1.91 times larger than the former. From these data we calculated that fully reconstructed glutamatergic synapses would have been labeled with 3.25 immunogold particles, while GABAergic synapses with 2.13 immunogold particles labeling NMDARs, which led to the unexpected conclusion that glutamatergic synapses contain only 1.6 times more NMDARs than GABAergic synapses.

Preembedding results clearly showed a purely postsynaptic localization of NMDARs in these synapses, nevertheless, we confirmed this also by measuring the distance of NMDAR immunogold labeling from the postsynaptic membrane. The distance distribution of gold particles measured perpendicularly from the postsynaptic membrane was very similar in GABAergic (median: 3.63 nm, interquartile range: −8.12–13.78 nm, n = 28) and glutamatergic synapses (median 7.26 nm, interquartile range −7.87–13.49 nm, n = 32, positive values refer to intracellular positions), confirming the postsynaptic origin of the immunogold labeling.

## Discussion

Here we investigated the subcellular distribution of NMDARs in the mouse hippocampus at the age, when GABA exerts depolarizing effects and the first synapse-driven synchronous network activity emerges (P6-7). Our major findings are that 1) NMDARs are associated with glutamatergic as well as GABAergic synapses, 2) GluN1, GluN2A and GluN2B subunits are all expressed postsynaptically in both glutamatergic and GABAergic synapses, 3) the density of NMDARs in glutamatergic synapses is about 3 times higher than in GABAergic synapses, and about 90 times higher than extrasynaptically and 4) since GABAergic synapses are larger, there is little difference in the NMDA receptor content of the two types of synapses at this age.

Co-expression of GABA_A_- and NMDA-receptors in GABAergic synapses during the first synapse-driven synchronous network events provide neuroanatomical evidence that the substrate of GABA_A_R-NMDAR cooperation in developing synapses could well be the GABAergic synapse. According to this model, GABA_A_Rs may facilitate the activation of NMDARs inside the same developing GABAergic synapse, the combined effect of which could be sufficient to depolarize even distant silent (only NMDAR-containing) glutamatergic synapses, and thereby allowing them to recruit AMPA receptors.

### NMDARs in Developmental Synchronous Network Activity

Spontaneous synchronous activity (SSA) is a fundamental feature of developing networks and indispensable for the formation and refinement of synapses [13], [30]. It is evolutionarily conserved, was shown to be present in primates including the human brain [21], [31], and can be observed throughout the mammalian central nervous system [2]. SSA-s are primarily dependent on synaptic transmission, the depolarizing actions of GABAergic transmission [32], [33], and the activation of NMDARs [2], [4], [6], [7].

The importance of NMDAR function during development is confirmed by vast amounts of experimental data. The lack of GluN1 subunit is lethal, mice without GluN2B subunit die within 1 day from birth [34]–[36], the blockade of NMDARs disrupts SSA-s both in the hippocampus and neocortex [6], [7], while its blockade only on postnatal day 7 reduces the number of neurons, mRNA levels of synaptophysin, and leads to behavioral dysfunction in the adult animals [12]. In addition, inhibition of NMDARs during development alters protein translation, disrupts synapse formation and hippocampus-dependent learning [11], for review see Bois et al, 2007 [37].

NMDARs are voltage and ligand gated ion channels, in addition to binding glutamate, they need coincident depolarization in order to get rid of the Mg^2+^-block, and take up the open conformation. In the adult brain, activation of AMPA receptors provides this depolarization. However, the first glutamatergic synapses are “silent’’, expressing only NMDARs, and incorporate AMPARs only later [15], [19], [38], [39]. During development, synaptogenesis is sequential, as GABAergic synapses are the first to be established, followed by glutamatergic ones [21], [22]. GABA, the main inhibitory neurotransmitter in the adult brain, exerts depolarizing actions during development, and is involved in the generation of SSA-s (reviewed in [18]). The depolarizing nature of GABAergic transmission is a universal feature of the developing CNS, and has also been shown in the human brain [40], [41]. The depolarizing effect of GABA is essential for the establishment of proper neuronal morphology and synapse formation *in vivo* [5], [20], [42] and provides the depolarization required for the removal of the Mg^2+^-block from NMDARs to drive SSA-s [4], [5], [43]. According to current views, the GABA_A_R-driven depolarization spreads to nearby glutamatergic synapses [4], [5], and leads to the activation of NMDARs and subsequent “AMPAficaton’’ [13], [15] of these synapses. However, our results suggest that NMDAR activation takes place first within the GABAergic synapses themselves.

### NMDA and GABA_A_ Receptor Cooperation within GABAergic Synapses during Early Development – Involvement in the Generation of Spontaneous Synchronous Activity

A large amount of data has been gathered during the past years on the developmental expression of NMDARs. GluN1 subunit mRNA and protein are present from early development, however the GluN2B/GluN2A ratio decreases during this period in rodent [24], [25], [28] and human brain [44]. The presence of GluN1 and GluN2 subunits in synapses was shown during development [19], furthermore GluN1 subunits are suggested to be expressed extrasynaptically during this period [27]. In spite of the growing mass of data, the exact subcellular distribution of NMDARs during development is still unknown and more importantly, the type of terminals that establish NMDAR containing synapses was not yet identified. We performed different combinations of labeling experiments, and found that GluN1, GluN2B and GluN2A subunits of the NMDARs were expressed exclusively postsynaptically in identified glutamatergic and GABAergic synapses. These data corroborate our previous study in which we showed the presence of NMDARs in GABAergic synapses of the adult hippocampus [23]. However, during development, these NMDARs have a different role, and are in a perfect position in the GABAergic synapses to respond to postsynaptic depolarization evoked by GABA. Our quantitative postembedding immunogold data demonstrated that NMDARs are enriched in glutamatergic and GABAergic synapses, while the former had only about 60% more NMDARs per synapse. This difference changes dramatically in the adult brain, where glutamatergic synapses contain 5 times more NMDARs than GABAergic ones [23]. The relatively higher level of NMDARs in GABAergic synapses during the first postnatal week suggests a fundamental role of these receptors in this location.

**Figure 5:**
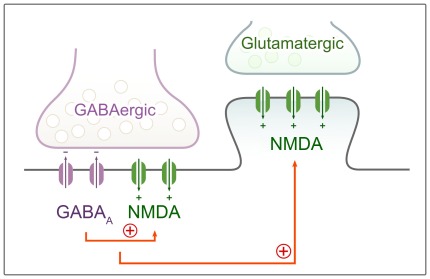
Schematic drawing shows the proposed mechanism for GABA_A_R and NMDAR cooperation during early postnatal development. Our results that GABA_A_ and NMDA receptors are co-expressed in GABAergic synapses can provide the anatomical basis for a new model of the generation of SSAs during the postnatal period. The GABA_A_R mediated current leads to postsynaptic depolarization in the GABAergic synapse that allows activation of NMDARs located in the same synapse. This homosynaptic receptor activation in GABAergic synapses causes strong local depolarization that leads to a subsequent heterosynaptic activation of NMDARs in otherwise silent (AMPA receptor-lacking) glutamatergic synapses. The schematic drawing illustrates the position of GABAAR and NMDAR at synapses, where their presence has already been proven by others or in our current study.

Our result that GABA_A_- and NMDA-receptors are co-expressed in GABAergic synapses can provide the anatomical basis for a new model of the initial generation of spontaneous synchronous activity during early development. We propose that the local depolarization in GABAergic synapses removes the Mg^2+^-block from postsynaptic NMDARs located in the very same synapses, and the subsequent Ca^2+^-influx, together with outward chloride currents initiates a strong local depolarization that spreads to nearby glutamatergic synapses, allowing the activation of those NMDARs as well (Fig.5). This mechanism would employ a homosynaptic cooperation between GABA_A_- and NMDA-receptors, and a subsequent heterosynaptic activation, instead of an exclusively heterosynaptic effect proposed before [4], [5]. Furthermore, Ca^2+^-influx via NMDARs in GABAergic synapses may also participate in synaptic plasticity mechanisms, and can contribute to the activity-dependent stabilization of these synapses during development.

### Possible Anchoring Mechanisms and the Source of Ligands for NMDARs in GABAergic Synapses

NMDARs are anchored to the postsynaptic density via PDZ-domain containing proteins in glutamatergic synapses. There are at least two PDZ-domain containing proteins present in the postsynaptic densities of GABAergic synapses, which could serve as anchoring sites for NMDARs. Synaptic scaffolding molecule (SSCAM) was found in GABAergic synapses [45], and was also shown to interact with NMDARs [46], [47]. Another PDZ-domain containing protein found in GABAergic synapses during development is glutamate receptor interacting protein 1 (GRIP1) [48]–[52]. Knocking out either of these two proteins is lethal, suggesting their important role from early development [53], [54].

Glutamate and aspartate are the most potent endogenous ligands of NMDARs. A possible source of NMDAR activation in GABAergic synapses could be glutamate spillover from neighboring glutamatergic synapses. However, there are multiple other possible sources, some of which being probably in a more favorable location to activate these NMDARs. Glutamate release from GABAergic terminals has been shown in the adult brain from vesicular glutamate transporter 3 (vGluT3) expressing vesicles [55], and vGluT3 is also expressed in the hippocampus during development [56]. Vesicular co-release of aspartate from GABAergic terminals has also been described in the hippocampus [57]. These results suggest that endogenous ligands released from GABAergic terminals could activate NMDARs in the same synapses. Other sources of ligands are also possible. The release of different neurotransmitters from axonal growth cones is well known [58], [59], and the release of glutamate from growth cones or astrocytes has also been described [60]–[64]. Furthermore, glutamate released from astrocytes can control synaptic strength [65], and influence GABAergic synaptic transmission [66]. Aguado and co-workers also showed that spontaneous Ca^2+^-transients could be observed in the astrocytic network during development, which is synchronous with neuronal SSA-s [67]. Thus, astrocyte-derived glutamate can also play a role in activating NMDARs in GABAergic synapses.

The expression levels of glial glutamate transporters were shown to be relatively low during postnatal development [68]. The level of extracellular glutamate using microdialysis probe was found to be between 0.4–1 µM in adult animals [69]–[71], and developmental levels were claimed to be similar [68]. Since glutamate activates NMDARs efficiently (EC_50_: 0.4–1.8 µM) [72], the ambient extracellular glutamate – derived from either of the above mentioned sources – should also be sufficient to activate NMDARs.

In our previous study, we showed that the retrograde nitric oxide signaling pathway could be activated via NMDARs in GABAergic synapses of the adult hippocampus [73], [23]. We also showed the presence of the nitric oxide signaling pathway in glutamatergic and GABAergic synapses of the developing brain, and the ability of this pathway to modulate synaptic transmission and to control SSAs during development [74]. Therefore, the enrichment of NMDARs in GABAergic synapses may not only be essential for the efficient induction of SSAs during development, but it is ideal for the local activation of this Ca^2+^-dependent retrograde nitric oxide-pathway.

## Materials and Methods

### Ethics Statement

All experiments were performed in accordance with the Institutional Ethical Codex and the Hungarian Act of Animal Care and Experimentation guidelines, which are in concert with the European Communities Council Directive of November 24, 1986 (86/609/EEC). The Animal Care and Experimentation Committee of the Institute of Experimental Medicine of Hungarian Academy of Sciences and the Animal Health and Food Control Station, Budapest, have specifically approved the experimental design under the number of 2302/003/FŐV/2006.

### Animals and Tissue Preparation

14 six- or seven-day-old male C57BL/6 mice were anesthetized with inhalation of isoflurane, followed by an intraperitoneal injection of 0.05–0.1 ml of an anesthetic mixture (containing 8.3 mg/ml ketamine, 1.7 mg/ml xylazin-hydrochloride, 0.8 mg/ml promethazinium-chloride). Animals were perfused transcardially with 0.9% NaCl solution for 1 minute, followed by a fixative for 40 minutes and finally 0.1 M PB for 10 minutes. The fixative contained 4% freshly depolymerized paraformaldehyde in 0.1 M phosphate buffer (PB) pH 7.4 for all experiments, except for the pre-embedding vGluT1-immunoperoxidase and GAD-immunoperoxidase single immunoreactions. The animals in the latter two reactions were perfused with a fixative containing 4% paraformaldehyde plus 0.25% glutar-aldehyde in PB. After perfusion, brains were removed and were not postfixed. Blocks containing the dorsal hippocampi were dissected and coronal sections were prepared on a vibratome (VT1200S, Leica, Germany) at 40–60 µm thickness for immunofluorescent experiments, 70–80 µm thickness for pre-embedding electron microscopy reactions and 300 µm thickness for freeze-substitution and low-temperature embedding. In all preembedding experiments, where we labeled NMDARs, the tissue was treated with pepsin for epitope retrieval – as described below. In other reactions, pepsin treatment was not performed.

### Primary Antibodies

For a summary of the antibodies used, see Table 1. We used a mouse monoclonal antibody raised against the amino acid residues 4-101 of GAD67 to label GABAergic terminals (Millipore, MAB5406, clone 1G10.2). The specificity of this antibody has been tested before [75]. To identify GABAergic terminals in the immunofluorescent and post-embedding experiments we used the GAD65/67 antibody No. 1440 raised in sheep [76], [77]. This antibody labels specifically the 65 and the 67 kD molecular weight isoforms of GAD [78]. To label glutamatergic terminals selectively, we used two antibodies. The first was a polyclonal guinea-pig antibody, raised against a synthetic peptide from rat vGluT1, which peptide does not overlap with the sequence of vGluT2; (Millipore, AB5905). The specificity of this antibody has also been described, and it gave identical staining with a well-characterized rabbit anti- vGluT1 antibody [79]. The other antibody for labeling glutamatergic terminals was a rabbit anti-vGluT1 antibody (Cat. No.: 135 303, aa 456–560, Synaptic Systems), which has also been characterized, and gave identical labeling with AB5905 [80].

**Table 1:** Antibodies used in the study.

Primary antibodies								
Raised in	Raised against	Protein conc.		Fluorescent	Preemb.	Postemb.	Source	Characterized
mouse	GAD67	1000 µg/mL		–	1∶3000	–	Millipore, MAB5406, clone: 1G10.2	Ref.: 75
sheep	GAD65/67	not available		1∶10000	–	1∶50	Oertel et al., Ab. No. 1440	Ref.: 76–78
guinea-pig	vGluT1	not available		1∶8000	1∶10000	–	Millipore, AB5905	Ref.: 79
rabbit	vGluT1	1000 µg/mL		–	–	1∶500	SynapticSystems, Cat. No.: 135 303	Ref.: 80
mouse	Bassoon	1000 µg/mL		1∶5000–1∶8000	–	–	Abcam, ab82958, clone: SAP7F407	Ref.: 81–83
rabbit	GluN1	720 µg/mL		1∶500	1∶400	1∶30	Prof. Masahiko Watanabe, C2-casette	Ref.: 29, 84, 86
rabbit	GluN2A	542 µg/mL		–	1∶400	–	Prof. Masahiko Watanabe, C-terminal	Ref.: 29, 84, 86
rabbit	GluN2B	264 µg/mL		–	1∶400	–	Prof. Masahiko Watanabe, C-terminal	Ref.: 25, 84–86
mouse	GluN1	1000 µg/mL		1∶500	–	–	Millipore, MAB363, clone: 54.1	Ref.: 87
guinea-pig	GABA_A_R β3	serum		1∶800	–	–	Prof. Ryuichi Shigemoto, C-terminal	Ref.: 88
rabbit	GABA_A_R γ2	1000 µg/mL		1∶4000	–	–	SynapticSystems, cat. no.: 224 003	See text.
Secondary antibodies								
Conjugated with			Raised in	Raised against	Dilution		Source	Molecule
DyLight 405			donkey	guinea-pig	1∶400		Invitrogen	whole IgG
Alexa 488			donkey	rabbit	1∶400		Invitrogen	whole IgG
Cy3			donkey	mouse	1∶1000		Invitrogen	whole IgG
Alexa 647– streptavidin (not antibody)					1∶1000		Invitrogen	streptavidin
biotin			donkey	sheep	1∶1000		Jackson Laboratories	whole IgG
biotin			donkey	guinea-pig	1∶200		Vector Laboratories	whole IgG
biotin			donkey	mouse	1∶200		Vector Laboratories	whole IgG
1.4 nm gold particle			goat	rabbit	1∶200		Nanoprobes	Fab-fragment
10 nm gold particle			goat	rabbit	1∶100		British Biocell International	whole IgG
6 nm gold particle			donkey	sheep	1∶100		Aurion	whole IgG

For the labeling of synapses, we used a mouse monoclonal antibody against Bassoon, a presynaptic cytoskeletal protein [81], which is present both in GABAergic and glutamatergic synapses [82]. This monoclonal antibody (Abcam, clone: SAP7F) was tested using transgenic knock-out (KO) mice lacking the Bassoon protein, and no labeling could be observed in the KO animal [83]. The specificity of the rabbit antibodies against the C-terminus of the NMDAR subunits GluN1, GluN2A and GluN2B had been well-characterized using immunoblot, antigen peptides and null mutant mice or conditioned knockout mice in both pre- and postembedding experiments [29], [84]–[86]. In the immunofluorescent experiments and double immunogold-immunoperoxidase reactions we used the same preembedding digestion protocol, as was used in the experiments for testing the specificity of the antibodies [86]. In addition, immunogold labeling against the different GluN1, GluN2A and GluN2B subunits displayed the same distribution in the tissue. We also used a mouse monoclonal antibody against the extracellular loop of the GluN1 subunit (Millipore, MAB363, clone: 54.1). The specificity of this antibody was proved using cortical pyramidal cell restricted GluN1 knockout animals [87]. To label GABA_A_-receptors, we used a guinea pig antibody raised against the beta3 subunit (produced and characterized in the laboratory of Ryuichi Shigemoto, Okazaki, Japan) [88], or a rabbit polyclonal antibody raised against the gamma2 subunit (SynapticSystems, raised against amino acids 39–67, cat. nr.: 224 003). The specificity of the latter antibody was confirmed by viral mediated focal deletion of GABA_A_R gamma2 subunit in mice (personal communication by László Acsády, Budapest, Hungary).

We extensively tested the possible cross-reactivity of the fluorescent secondary and gold conjugated secondary antibodies used in multiple labeling experiments. No cross-reactivity was found in any of the cases. Selective labeling, resembling that obtained with the specific antibodies, could not be detected if primary antibodies were omitted. In the post-embedding control reactions unspecific binding of gold conjugated secondary antibodies was totally absent without primary antibodies. In the pre-embedding control reactions the 1.4 nm gold conjugated goat anti-rabbit Fab-fragment (NanoProbes) was tested without primary antibodies. This secondary antibody gave a linear density of only 0.006 gold particles/µm, when applied at 1∶100 dilution. This is two orders of magnitude smaller, than the lowest detected synaptic immunogold density.

### Immunofluorescent Labeling and Confocal Laser-scanning Microscopy

Before the immunofluorescent stainings, the sections were washed in PB. For synaptic detection of NMDARs, pretreatment with pepsin is essential [86], thus sections were incubated in 0.2 M HCl solution containing 1 mg/ml pepsin (Dako) at 37C° for 2–5 minutes in all immunofluorescent experiments. This was followed by washes in PB and tris-buffered saline (TBS) and blocking for 1 hour in 1% human serum albumin (HSA) (Sigma) dissolved in TBS. After this, sections were incubated in mixtures of primary antibodies overnight at room temperature. In the quadruple-labeling experiments the primary antibodies were the guinea-pig anti-vGluT1 (1∶8000), the sheep anti-GAD65/67 (1∶10000), the mouse anti-Bassoon (1∶8000) and the rabbit anti-GluN1 (1∶400) antibodies, diluted in TBS. In the experiments for the direct colocalization of NMDARs and GABA_A_Rs a triple- and a double-labeling reaction was performed. In the triple reaction, a mixture of the following primary antibodies was used: the mouse anti-Bassoon (1∶5000), the rabbit anti-GluN1 (1∶500) and the guinea pig anti-beta3 subunit of GABA_A_ receptor (1∶800). In the double reaction we used the mouse anti-GluN1 (1∶500) and the rabbit anti-gamma2 subunit of GABA_A_-receptor (1∶4000). After incubation in primary antibody mixtures, sections of the quadruple reaction were washed in TBS and incubated for 4 hours in biotinylated donkey anti-sheep antibody (1∶1000, Jackson Laboratories) diluted in TBS. After subsequent washes in TBS, sections were incubated for 4 h in the mixture of the following secondary antibodies: DyLight 405 conjugated donkey-anti-guinea-pig (1∶400, Invitrogen), Alexa488 conjugated donkey-anti-rabbit (1∶400, Invitrogen), Cy3 conjugated donkey-anti-mouse (1∶1000, Invitrogen), and Alexa647 conjugated streptavidin (1∶1000, Invitrogen). In the triple reaction, the secondary antibodies were: DyLight 405 conjugated donkey-anti-guinea-pig (1∶400), Alexa488 conjugated donkey-anti-rabbit (1∶400), Cy3 conjugated donkey-anti-mouse (1∶1000), and in the double reaction: Alexa488 conjugated donkey-anti-rabbit (1∶400), Cy3 conjugated donkey-anti-mouse (1∶1000). Secondary antibody incubation was followed by washes in TBS, PB, and the sections were mounted onto glass slides, coverslipped with Aqua-Poly/Mount (Polysciences). Immunofluorescence was analyzed using a Nikon Eclipse Ti-E inverted microscope (Nikon Instruments Europe B.V., Amsterdam, The Netherlands), with a CFI Plan Apochromat VC 60×H oil immersion objective (numerical aperture: 1.4) and an A1R laser confocal system. We used 405, 488, 561 and 642 nm lasers (CVI Melles Griot), and scanning was done in line serial mode. Image stacks were obtained with NIS-Elements AR software, and deconvolved using Huygens Professional software (www.svi.nl).

### Pre-embedding Immunoelectron-microscopy

We performed single immunoperoxidase experiments to measure the sizes of glutamatergic and GABAergic synapses. Only for these reactions, we used strongly fixed sections (4% paraformaldehyde plus 0.25% glutaraldehyde) and washed them in PB and TBS. These sections were not treated with pepsin. Next, we blocked them in 1% HSA diluted in TBS for 1 h, and incubated them with either anti-vGluT1 (1∶10000) or mouse anti-GAD67 (1∶3000) diluted in TBS for 48 hours. This was followed by washes in TBS, and a 24 h incubation, either with biotinylated donkey anti-guinea pig (1∶200, Vector Laboratories) or biotinylated donkey anti-mouse (1∶200, Jackson Laboratories) diluted in TBS. Next, we washed the sections in TBS, and incubated them in Elite ABC (1∶300, Vector Laboratories) diluted in TBS. After this, sections were washed in TBS, and tris-buffer (TB) pH 7.6. The immunoperoxidase reaction was developed using 3,3-diaminobenzidine (DAB, Sigma-Aldrich) as chromogen. After subsequent washes, sections were osmified and processed for dehydration.

For combined immunogold-immunoperoxidase stainings we used 4% paraformaldehyde fixed tissue. Sections were washed in PB, treated with pepsin as described above, and further washed in PB and TBS. After this, we incubated the sections in 1% HSA diluted in TBS. Then the sections were incubated for 48 hours in the following solutions of primary antibodies diluted in TBS: either guinea-pig anti-vGluT1 (1∶10000) or mouse anti-GAD67 (1∶3000) was mixed with rabbit anti-GluN1 (1∶400), or rabbit anti-GluN2B (1∶400), or rabbit anti-GluN2A (1∶400). After repeated washes in TBS, sections were treated with blocking solution (Gel-BS) containing 0.5% cold water fish skin gelatin (GE Healthcare, Little Chalfont, UK) and 0.5% HSA in TBS for 1 h. This was followed by 24 h incubation in the following solutions of secondary antibodies diluted in Gel-BS: 1.4 nm gold conjugated goat anti-rabbit Fab-fragment (1∶200, NanoProbes) combined either with biotinylated donkey anti-guinea pig (1∶200, Vector Laboratories) or biotinylated donkey anti-mouse (1∶200, Jackson Laboratories). After intensive washes in TBS and 0.1 M PB sections were treated with 2% glutar-aldehyde in 0.1 M PB for 15 minutes to fix the gold particles into the tissue. This was followed by further washes in 0.1 M PB, TBS and incubation in Elite ABC (1∶300, Vector Laboratories) diluted in TBS. After this, sections were washed in TBS, and tris-buffer (TB) pH 7.6. The immunoperoxidase reaction was developed using 3,3-diaminobenzidine (DAB, Sigma-Aldrich) as chromogen. After repeated washes in TBS and enhancement conditioning solution (ECS, Aurion), gold particles were intensified using the silver enhancement solution (SE-EM, Aurion) for 40–60 minutes at room temperature. After subsequent washes, sections were treated with 0.5% osmium-tetroxide in PB for 20 minutes. Then sections were dehydrated in ascending ethanol series and acetonitrile, and embedded in epoxy resin (Durcupan, ACM, Fluka, Buchs, Switzerland). During dehydration sections were treated with 1% uranyl-acetate in 70% ethanol for 20 minutes. For the electron microscopic investigations, resin infiltrated hippocampi were placed into silicone moulds, and cured. After polymerization, 80–100 nm thick sections were cut using a Leica EM UC6 ultramicrotome (Nussloch, Germany), and picked up on formvar-coated single-slot copper grids. The sections were examined using a Hitachi H-7100 electron microscope (Tokyo, Japan) and a side-mounted Veleta CCD camera (Olympus Soft Imaging Solutions).

### Post-embedding Immunoelectron-microscopy

For Lowicryl embedding the same procedure was used as described previously [89]–[91]. Briefly, after washing in PB, the 300 µm thick sections from 4% paraformaldehyde fixed hippocampi were transferred into sucrose solutions in 0.1 M PB for cryoprotection. After slamming onto gold-plated copper blocks cooled in liquid nitrogen, low temperature dehydration, and freeze-substitution, the sections were embedded in Lowicryl HM20 resin (Chemische Werke Lowi). Postembedding immunohistochemistry was performed on 70 nm thick sections of Lowicryl-embedded hippocampi from two mice. The sections were picked up on formvar-coated single slot nickel grids, allowing immunoreaction on one side of the sections. These reactions were used to compare NMDAR expression between GABAergic and glutamatergic synapses by colocalizing the GluN1 subunit either with GAD65/67 or vGluT1, using the mirror technique. Long, continuous series of sections were picked up on grids in a way, that synapses cut in halves could be treated with different antibody solutions on adjacent sections. Grids were incubated on drops of blocking solution for 1 h, followed by incubation on drops of primary antibodies overnight. The blocking solution, which was also used for diluting the primary and secondary antibodies, contained 2% HSA and 0.03% Triton X-100 in TBS. Three adjacent grids were used, the first grid was reacted against the rabbit anti-vGluT1 (1∶500), the second grid was reacted against rabbit anti-GluN1 (1∶30), and the third grid was reacted against sheep anti-GAD65/67 (1∶50). This way, we could measure the NMDAR content of identified GABAergic and glutamatergic synapses on the very same grids. After incubation in primary antibodies for 18 h, sections were washed in TBS and incubated for 5 h on drops of secondary antibodies diluted in blocking solution, which contained polyethylene-glycol (0.5 mg/ml). We used 10 nm gold particle conjugated goat anti-rabbit (1∶100; British Biocell International), and 6 nm gold particle conjugated donkey anti-sheep (1∶100; Aurion) secondary antibodies. The 6 nm gold particles were intensified using silver enhancement solution (SE-EM; Aurion) for 35 min at room temperature. After several washes, sections were rinsed in ultrapure water and contrasted with saturated aqueous uranyl acetate and lead citrate. The sections were examined using a Hitachi H-7100 electron microscope (Tokyo, Japan) and a side-mounted Veleta CCD camera (Olympus Soft Imaging Solutions).

### Analysis

When data populations in this work had a Gaussian distribution according to the Shapiro-Wilks W test, we reported parametric statistical features (mean ± SD), otherwise we reported non-parametric statistical features (median, interquartile range). All examined synapses in this work were sampled from the stratum radiatum of the hippocampal CA1 area. The sizes of glutamatergic and GABAergic synapses were measured from serial sections of fully reconstructed synapses. The length between the edges of anatomically defined synapses was measured on subsequent images of the sampled synapses, and the sum of the measured lengths from each synapse was multiplied by the section thickness. For the semi-quantitative analyses of immunogold particles for NMDAR subunits in the pre-embedding experiments, we counted gold particles within the defined GABAergic and glutamatergic synapses and along the extrasynaptic membrane. Immunogold particles were considered to be membrane or synapse-associated, if they were not further away from the membrane than 40 nm. In the post-embedding immunogold reactions, 40 nanometer-wide bands were chosen on the two sides of the synaptic membrane as an area representing membrane-associated gold particle labeling. The reason for choosing 40 nm bands is that the epitope (the C-terminus of the NMDAR) is located a few nanometers intracellularly, the length of the primary and secondary IgG antibody molecules are ∼15 nm each, and the radius of gold particle is 5 nm. According to our experience, gold particles may also move a few nanometers on the surface of the section after immunohistochemistry, which can also cause a few nanometers of shift in receptor labeling. However, this distribution was also tested in postembedding reactions and we found that actually the majority of all gold particles were within two 20-nm-wide bands from the postsynaptic membrane in both GABAergic and glutamatergic synapses (median: 3.63 nm, interquartile range: −8.12–13.78 nm; median 7.26 nm, interquartile range −7.87–13.49 nm, respectively). For the measurements, we used open-source image analyzing software: ImageJ/Fiji.
